# Supplementary figures and images for: Frequency of and factors associated with antiseizure medication discontinuation discussions and decisions in patients with epilepsy: A multicenter retrospective chart review
Source: Epilepsia Open. 2023 Feb 14;8(2):371–85. doi: 10.1002/epi4.12695 (PMC10235583; doi:10.1002/epi4.12695)

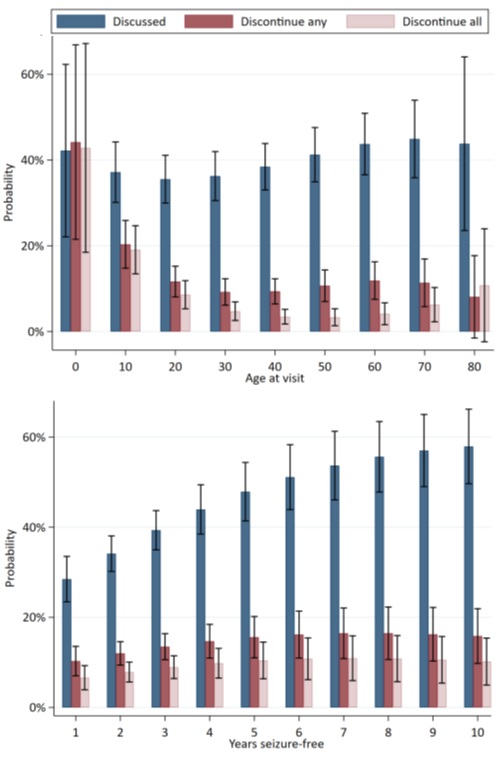

Supplement: Supplementary file 1 — Figure S1: [file EPI4-8-371-s001.jpg]

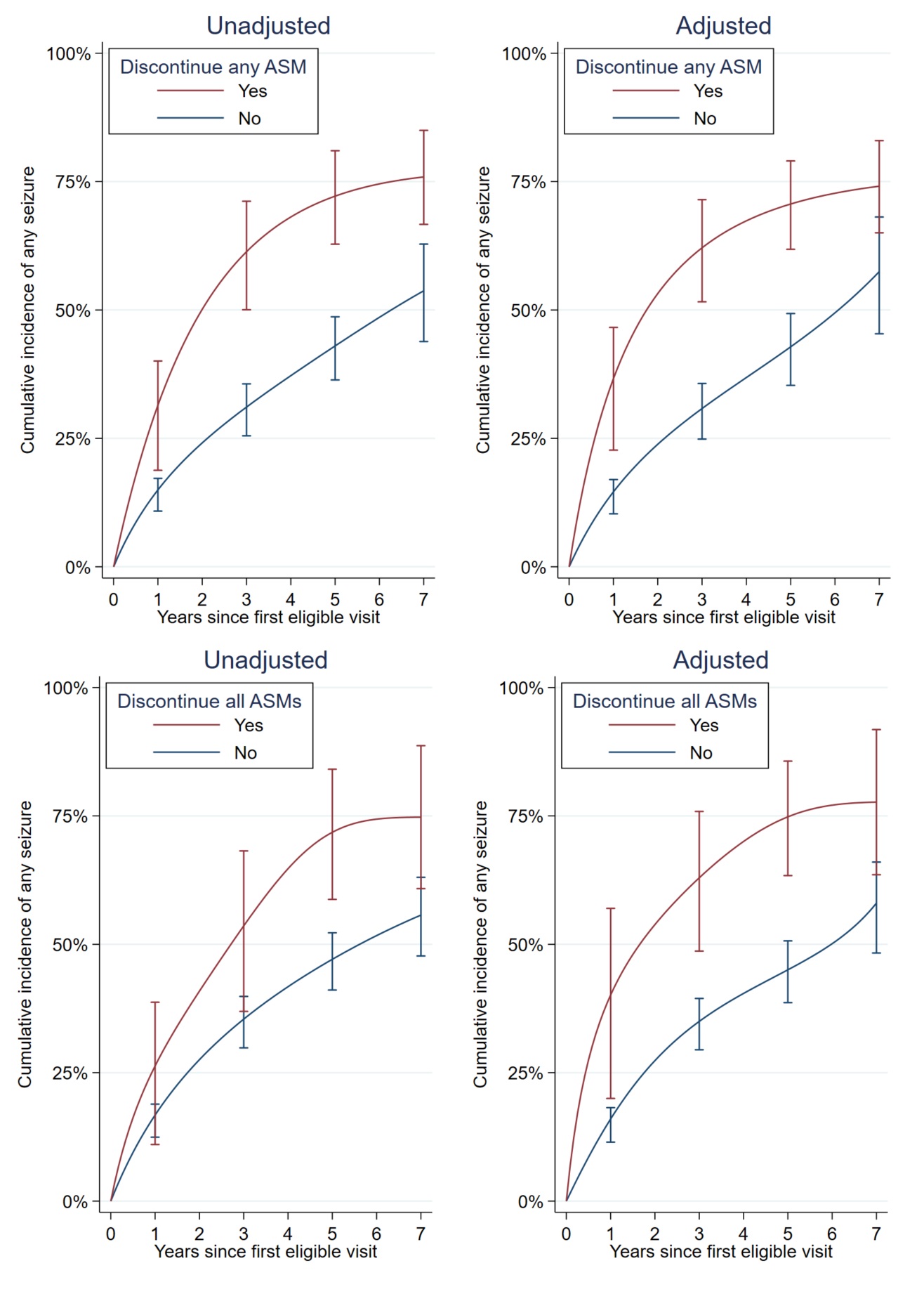

Supplement: Supplementary file 2 — Figure S2: [file EPI4-8-371-s003.jpg]
