# Supplementary material for: Frequency of and factors associated with antiseizure medication discontinuation discussions and decisions in patients with epilepsy: A multicenter retrospective chart review
Source: Epilepsia Open. 2023 Feb 14;8(2):371–85. doi: 10.1002/epi4.12695 (PMC10235583; doi:10.1002/epi4.12695)
Supplement: Supplementary file 3 — Table S1: [file EPI4-8-371-s002.docx]

**Supplemental Table 1:** Testing for multicollinearity in our ‘per-visit’ models. A common rule of thumb is that multicollinearity is problematic when variance inflation factors exceed 10. All variance inflation factors were less than or equal to 2.5.

| **Variable** | **Variance inflation factor** |
| --- | --- |
| Age | 2.5 |
| Duration epilepsy | 2.2 |
| Site: UM | 1.9 |
| Site: SEIN | 1.9 |
| Epileptologist | 1.8 |
| Greater than 9 seizures | 1.5 |
| Developmental delay | 1.5 |
| ASM number | 1.5 |
| Genetic | 1.5 |
| VNS | 1.4 |
| Older generation ASM | 1.4 |
| Self-limited syndrome | 1.4 |
| Focal epilepsy | 1.3 |
| Structural | 1.3 |
| Duration seizure-free | 1.3 |
| Prior discontinuation attempt | 1.3 |
| MD | 1.3 |
| Epileptiform EEG | 1.2 |
| Status epilepticus | 1.2 |
| Female | 1.2 |
| Impairing awareness | 1.2 |
| Motor | 1.2 |
| White | 1.2 |
| Infectious | 1.2 |
| Family history | 1.1 |
| Febrile seizures | 1.1 |
| Metabolic | 1.1 |
| Immune | 1.0 |

UM: University of Michigan; SEIN: Stichting Epilepsie Instellingen Nederland; ASM: antiseizure medication; VNS: vagal nerve stimulator; MD: medical doctor.

**Supplemental Table 2**: Probability of discontinuation discussions and planning to discontinue at least one antiseizure medication (ASM) according to being below versus above the legal driving age at the time of the first visit in our observation period (≥16 years in the US; ≥18 years in the Netherlands). Each visit represented an observation.

|  |  | **Below driving age** | **Above driving age** | **p-value** |
| --- | --- | --- | --- | --- |
| Discussion | Unadjusted | 39% (32%-46%) | 39% (35%-43%) | 0.99 |
|  | Adjusted | 44% (35%-54%) | 40% (35%-44%) | 0.43 |
| Discontinue | Unadjusted | 21% (15%-26%) | 10% (8%-13%) | **<0.01** |
| any ASM | Adjusted | 19% (11%-27%) | 13% (9%-16%) | 0.16 |
| Discontinue | Unadjusted | 19% (13%-24%) | 5% (3%-7%) | **<0.01** |
| all ASMs | Adjusted | 13% (8%-18%) | 8% (4%-11%) | 0.14 |

**Supplemental Table 3**: Percent variation in each outcome due to provider-to-provider differences. Each visit represented an observation. Each number displays the intraclass correlation coefficient (ICC) and 95% confidence interval from multilevel logistic regressions where the outcome was either discussion (top row) or discontinuation (bottom row), with a random intercept for each provider. We adjusted for all variables listed in Table 2. Provider characteristics were whether the provider is an MD versus physician extender, and epileptologist versus non-epileptologist.

|  | **ICC, unadjusted** | **ICC, adjusted for patient characteristics** | **ICC, adjusted for patient + provider characteristics** |
| --- | --- | --- | --- |
| **Discussion** | 12% (6%-22%) | 11% (5%-23%) | 11% (5%-22%) |
| **Discontinue any ASM** | 20% (10%-37%) | 19% (9%-37%) | 18% (8%-35%) |
| **Discontinue all ASMs** | 26% (12%-46%) | 14% (4%-38%) | 13% (8%-38%) |

ASM: antiseizure medication

**Methods Supplement**

We calculated seizure relapse curves as follows. First, we assessed the date of the patient’s first visit (beginning follow-up 1/2015), the date when each patient decided to discontinue if applicable, the date of the patient’s first seizure prior to January 1, 2022, if any, and the date of the patient’s last follow-up visit prior to January 1, 2022. We included one observation for each person-month of follow-up. The main predictor was whether the patient decided to discontinue ASMs, which started out as “no,” until the first instance of deciding to discontinue if applicable, after which this variable changed to “yes” for the remainder of follow-up. We chose ‘planned discontinuation’ as the main predictor rather than ‘completion of discontinuation,’ given ‘completion’ would have suffered from immortal time bias (only the lowest risk patients complete tapering without having a seizure). Patients were censored upon their first seizure after their first eligible visit, their last follow-up visit, or January 1, 2022, whichever came first. We then calculated an unadjusted parametric survival curve from a discrete time logistic model, then a standardized survival curve adjusted for all covariates listed in Table 2 in accordance with best practices.^40^ We calculated cumulative incidence as 1 minus the survival curve at each timepoint. We obtained confidence intervals via 1,000 bootstrapped replications. This approach provided several key advantages: 1) encoded tapering as a time-varying covariate (to avoid misattributing pre-discontinuation time to the “discontinuation” group), 2) avoided requiring the proportional hazards assumption which applies to Cox models but not discrete time logistics models, and 3) adjusted survival curves which otherwise would not have been possible if we had simply drawn Kaplan-Meier curves.

Reference:

40. Hernán MA. The hazards of hazard ratios. *Epidemiology*. 2010; 21(1):13–5.
